# TSG6 impacts the immune microenvironment and drug sensitivity in gastric cancer

**DOI:** 10.1590/1806-9282.20250176

**Published:** 2025-07-07

**Authors:** Valbert Oliveira Costa, Pedro Robson Costa Passos

**Affiliations:** 1 Universidade Federal do Ceará, Center for Research and Drug Development – Fortaleza (CE), Brazil.

Dear Editor,

We were very pleased to read the study by Durak et al.^1^, in which they revealed the significant role of high hyaluronan levels and increased *TNFAIP6* (TSG-6) mRNA expression in the pathobiology of gastric cancer. This study provides interesting insights into the molecular mechanisms underlying extracellular matrix abnormalities in gastric cancer. Inspired by their findings, we performed additional analyses focused on TSG-6 to further validate and expand upon the study's conclusions.

Initially, we analyzed transcriptomic datasets containing sequencing data from normal gastric tissues and gastric cancer tissues. Using a Wilcoxon rank-sum test, we observed statistically significantly higher expression levels of TSG-6 in tumor samples compared to normal samples in the Cancer Genome Atlas Cohort (TCGA-STAD) (Figure 1) (p<0.001) and GSE29272 cohort (p<0.001). This result corroborates the findings of Durak et al.^1^ in larger cohorts through alternative gene expression platforms, reinforcing TSG-6 as a key component in the pathogenesis of gastric cancer.

**Figure 1:**
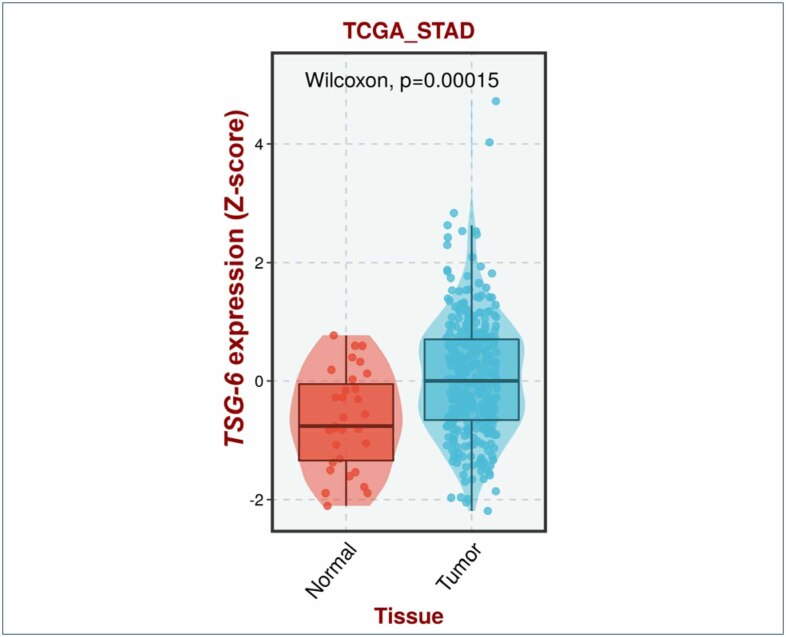
Box-plot comparing *TSG-6* expression between normal tissue and tumor tissue.

Next, we utilized TIMER^2^, a computational algorithm to estimate immune cell infiltration, across patients from the gastric cancer transcriptomic cohort (TCGA-STAD). Our analyses through Spearman's rank correlation coefficient revealed a correlation between elevated TSG-6 expression and infiltration of immune cells, particularly neutrophils (Cor=0.51, p<0.001), macrophages (Cor=0.27, p<0.001), and CD8+ T lymphocytes (Cor=0.24, p<0.001). Our findings suggest that TSG-6 influences the immune landscape within the gastric tumor microenvironment.

Using the TCGA cohort, we stratified patients into two groups based on TSG-6 expression: TSG-6-high (>median) and TSG-6-low (Imedian). Gene set enrichment analysis demonstrated that the TSG-6-high group was significantly associated with enhanced inflammatory responses, increased tumor necrosis factor-α (TNF-α) signaling via nuclear factor (NF)-κB, and heightened epithelial–mesenchymal transition activity (Figure 2). These results further underscore the multifaceted role of TSG-6 in gastric cancer, suggesting its involvement in both immune regulation and tumor aggressiveness.

**Figure 2:**
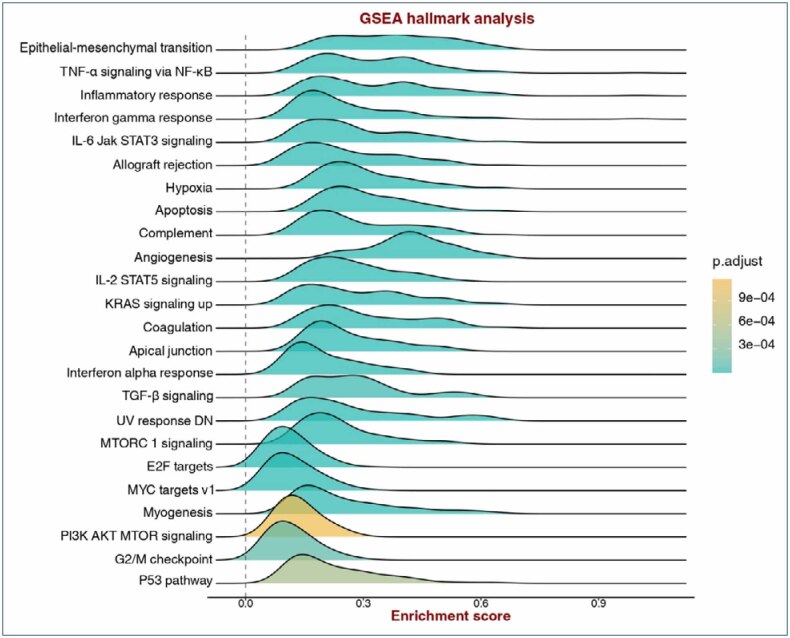
Gene set enrichment analysis comparing tumors with high *TSG-6* expression to those with low *TSG-6* expression.

Considering the distinct tumor biology associated with different TSG-6 expression groups, we conducted a drug sensitivity analysis using data from cell lines treated with various compounds via the BEST tool^3^. Specifically, we assessed whether TSG-6 expression correlated with the half-maximal inhibitory concentration (IC50) values of anticancer drugs across 16 transcriptomic datasets from gastric cancer patients, leveraging data from GDSC1 and GDSC2^4^ (Figure 3).

**Figure 3:**
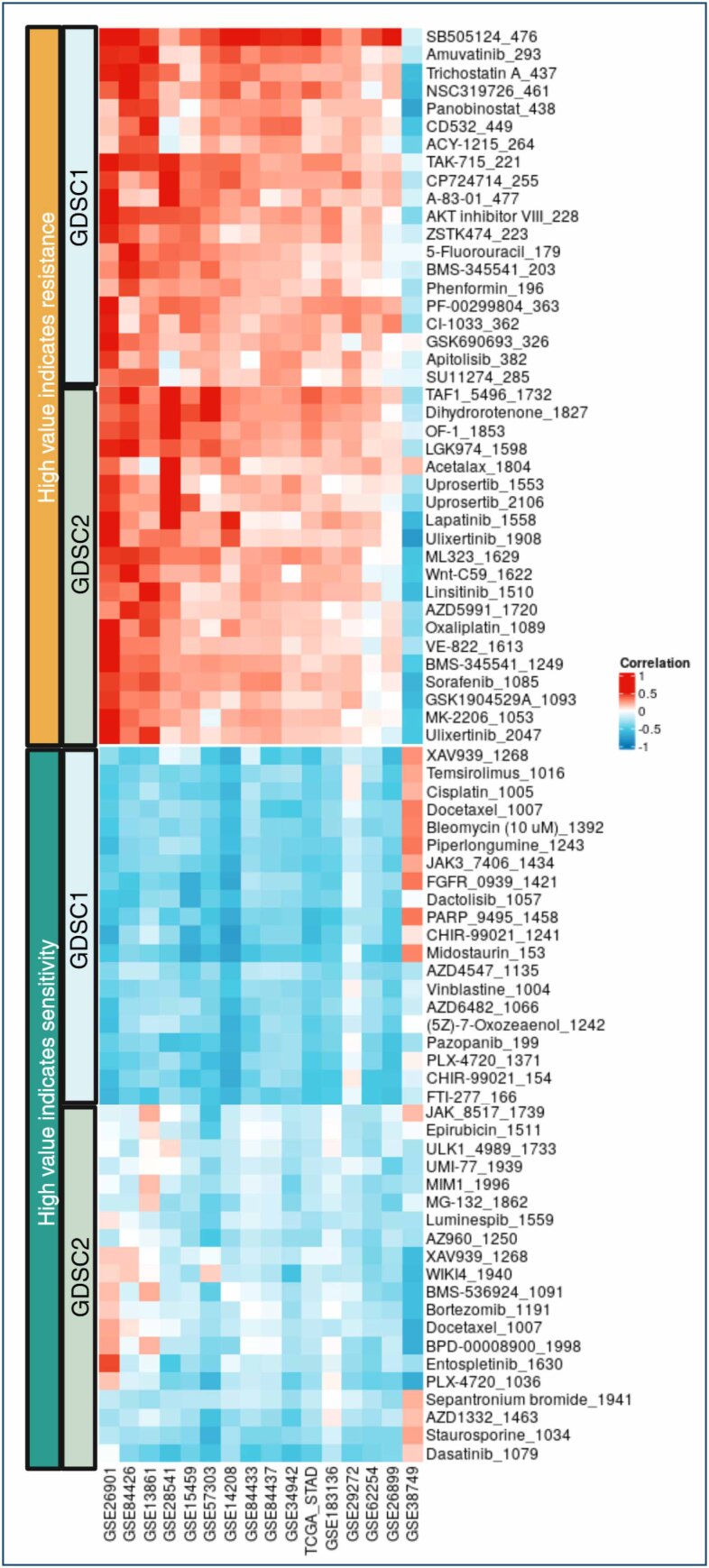
Correlation of *TSG-6* expression with drug sensitivity across compounds on GDSC1 and GDSC2 datasets.

Interestingly, higher TSG-6 expression levels were associated with increased resistance to 5-fluorouracil, oxaliplatin, and sorafenib. Conversely, elevated TSG-6 levels correlated with decreased resistance to cisplatin, docetaxel, and epirubicin. These findings suggest that TSG-6 may serve as a predictive biomarker for therapeutic response in gastric cancer, potentially guiding treatment decisions. For instance, patients with a high TSG-6 expression might benefit from substituting oxaliplatin with cisplatin to enhance treatment efficacy. This highlights the potential clinical relevance of TSG-6 not only in understanding tumor biology but also in optimizing personalized therapeutic strategies for gastric cancer.

In summary, our findings validate those of Durak et al.^1^, highlighting the role of TSG-6 in gastric cancer pathogenesis and its association with immune infiltration and therapeutic sensitivity. We recommend that future studies further investigate TSG-6 as a biomarker to guide personalized treatment strategies, given its potential to predict drug sensitivity and resistance.

## Data Availability

The datasets generated and/or analyzed during the current study are available from the corresponding author upon reasonable request.
